# Effect of *GATA3* rs3824662 gene polymorphism in Han Chinese children with pre-B-cell acute lymphoblastic leukemia with 10 years follow-up

**DOI:** 10.3389/fped.2022.1044866

**Published:** 2023-01-11

**Authors:** Xinran Chu, Maoxiang Qian, Jin Yang, Dong Wu, Jing Gao, Lu Cao, Fang Fang, Jian Pan, Hui Zhang, Shaoyan Hu

**Affiliations:** ^1^ Department of Hematology and Oncology, Children's Hospital of Soochow University, Suzhou, China; ^2^ Department of Hematology and Oncology, Institute of Biomedical Sciences, Children's Hospital of Fudan University, Shanghai, China; ^3^ Department of Pediatrics, Subei People's Hospital of Jiangsu Province, Yangzhou, China; ^4^ Department of Pediatrics, Yiyuan People’s Hospital, Zibo, China; ^5^ Department of Emergency, Children’s Hospital of Soochow University, Suzhou, China; ^6^ Institute of Pediatric Research, Children’s Hospital of Soochow University, Suzhou, China.; ^7^ Department of Hematology and Oncology, Fujian Branch of Shanghai Children’s Medical Center, Fujian Children’s Hospital, Fuzhou, China; ^8^ Department of Hematology and Oncology, Shanghai Children’s Medical Center, Shanghai Jiao Tong University School of Medicine, Shanghai, China

**Keywords:** *GATA3* rs3824662, pre-B-cell acute lymphoblastic leukemia, pediatric, early treatment, sepsis

## Abstract

**Purpose:**

To evaluate the influence of *GATA3* rs3824662 on pre-B-cell acute lymphoblastic leukemia (pre-B-cell ALL) susceptibility and long-term prognosis in Han Chinese children with pre-B-cell ALL treated with the CCLG-2008 protocol at the Children’s Hospital of Soochow University.

**Methods:**

A total of 256 patients with childhood pre-B-cell ALL under the CCLG-2008 protocol were enrolled in this study, and 174 healthy children were used as case controls. *GATA3* rs3824662 genotyping was performed using a polymerase chain reaction, followed by Sanger sequencing. The association of genotype with clinical characteristics, treatment response, adverse events, and outcomes were analyzed.

**Results:**

The A allele frequency of *GATA3* rs3824662 in patients with pre-B cell ALL was significantly higher than that in healthy children (OR = 1.41, 95% CI = 1.042–1.908; *P *= 0.026). Among patients with pre-B-cell ALL, the *GATA3* rs3824662 AA genotype was associated with poor prednisolone response and high blast cell burden on day 15 of the induction therapy (*P *= 0.011 and 0.007, respectively). Patients with the rs3824662 AA variant suffered more episodes of sepsis than those with the CC or CA variants (*P *= 0.021). The GATA3 rs3824662 AA genotype was significantly associated with sepsis [hazard ratio (HR) = 3.375; *P *= 0.01]. No significant differences were found in the cumulative incidence of relapse, overall survival, and event-free survival among all genotypes.

**Conclusion:**

*GATA3* rs3824662 was associated with susceptibility in Han Chinese children with pre-B-cell ALL and could be a possible risk factor for poor early treatment response and treatment-related sepsis.

## Introduction

The occurrence of leukemia is believed to be influenced by both environmental and genetic factors. Inherited genetic variation strongly influences both susceptibility and treatment outcomes of childhood acute lymphoblastic leukemia (ALL). Over the past few years, large-scale genome-wide association studies have identified a few susceptibility genes for pediatric ALL, including *IKZF1*, *CEBPE*, *PIP4K2A*, *CDKN2A*, *TP63*, and *GATA3* (1, 2)*.* The association between *GATA3* rs3824662 and B-cell ALL susceptibility was first identified among Hispanic people in 2013 (3). Acute lymphoblastic leukemia susceptibility has been confirmed by multiple studies among different ethnicities. Recent reports have shown that the rs3824662 A allele is associated with a high initial white blood cell (WBC) count, old age, and Ph-like ALL profiling (4–6). In addition, the risk allele A confers high minimal residual disease (MRD) levels by the end of induction therapy and an increased risk of recurrence *via* JAK-STAT-autophagy activation, demonstrating its critical role in ALL pathogenesis and prognosis (5–7).

Immunosuppression has been demonstrated to be an unavoidable accompanied side effect during leukemia therapy. However, immune reconstitution in leukemia therapy depends on the type of lymphocytes, residual thymic function, treatment intensity, and individual genetic factors (8). *GATA3* has been regarded as a master regulator in both innate and adaptive immunity because of its crucial role in hematopoietic cells. The function of *GATA3* as a key regulator in T cell development, differentiation, and maintenance, especially for T helper 2 (Th2) cells, is well established (9–11). The rs3824662 locus is located in the enhancer region of *GATA3*, leading to increased *GATA3* transcript levels (7, 12). However, the association between rs3824662 and chemotherapy-related adverse events has not yet been investigated.

With this in mind, we systemically analyzed the association between *GATA3* rs3824662 and clinical features and found that *GATA3* rs3824662 was associated with susceptibility in Chinese children of Han nationality with pre-B-cell ALL and poor early treatment response. In addition, patients with the AA variant had more episodes of sepsis than those with the CC or CA genotypes.

## Patients and methods

### Patients

A total of 256 newly diagnosed children with pre-B-cell ALL and 174 healthy controls were enrolled in this study between November 2012 and August 2015 at the Children's Hospital of Soochow University. All patients were treated according to the Chinese Children Leukemia Group-ALL 2008 (CCLG-ALL 2008) protocol (13). Informed consent forms were signed, and the study was conducted following the principles of the Declaration of Helsinki. The characteristics of patients were presented in Supplementary Table S1.

### Response evaluation

The National Comprehensive Cancer Network guidelines version 1.2021 on ALL was used to assess treatment response. Briefly, poor prednisolone response was defined as an absolute count of leukemia cells ≥1,000/µl in the peripheral blood after induction therapy with prednisone for seven consecutive days. The bone marrow morphology was categorized as M1 (bone marrow blasts <5%), M2 (≥5% and <25%), and M3 (≥25%). Complete remission (CR) was defined as no symptoms of leukemia cell infiltration in the body, no circulating blasts after induction therapy, and M1 in the bone marrow, with normal levels of platelets and absolute neutrophil counts. Induction failure was defined as failure to achieve CR at the end of induction. Relapse was defined as primitive and naive lymphocytes >20% in the bone marrow or any extramedullary site (central nervous system, testis, or skin) after CR or primary or naive lymphocytes >5%–20% in the bone marrow associated with evidence of a positive conversion in molecular biology. The stages of relapse were divided into three stages: very early relapse (recurrence <18 months from initial diagnosis), early relapse (recurrence ≥18 months but <36 months from initial diagnosis), and late relapse (recurrence from initial diagnosis ≥36 months). Overall survival (OS) calculation started from the date of diagnosis and ended at the date of death. Missed follow-up data were censored at the last follow-up. Event-free survival (EFS) was defined as the time interval between the date of achieving CR and recurrence or death. Relapse-free survival (RFS) was calculated from the date of CR achievement to the date of relapse.

### Adverse side effects

The adverse effects of CCLG-2008 therapy were evaluated and graded using the Common Terminology Criteria for Adverse Events 5.0. Sepsis, myelosuppression, pneumonia, and liver function impairment were included in the analysis of adverse effects.

### Genotyping

Genomic DNA was extracted from the peripheral blood of patients in the CR stage or healthy controls using the QIAamp DNA Mini Kit (QIAGEN, Germany) and stored at −20 °C until use. The touchdown polymerase chain reaction was used to amplify the intron fragment of rs3824662. The corresponding DNA fragments were amplified by a polymerase chain reaction using the following primers: forward, 5′-TATCACCCTCCCCACCA-3′; reverse, 5′-GGAAAGCCCCAGATCAA-3′, and then sent for sequencing (Suzhou Jinweizhi Biotechnology Co., Ltd.). Mutation Surveyor V4.0.8 software was used to analyze the SNP genotypes.

### Statistical analysis

SPSS statistics 22 and GraphPad Prism 5 were used to analyze the data. The chi-square test was used for the comparison of qualitative data (frequency and percentage) and the Hardy–Weinberg equilibrium test. Logistic regression analysis was used to perform the susceptibility analysis. The association between genotype and the number of adverse events was based on the Kruskal–Wallis test. Survival analysis was performed using the Kaplan–Meier method. The log-rank test was used to compare survival among groups. All *P*-values were obtained from two-sided tests, and statistical significance was set at *P* < 0.05.

## Results

### Baseline characteristics of patients with pre-B-cell ALL

The baseline characteristics of the patients with pre-B-cell ALL were summarized in Supplementary Table S1. The last follow-up period was August 2022, and the median follow-up period was 100.32 months. Among the 256 patients with pre-B-cell ALL, 108 (42.2%) were female and 148 (57.8%) were male; the male/female ratio was 1.4:1. There were 38 (14.8%) patients who were older than 10 years old, and 54 (21.1%) had high WBC (>50 × 10^9^/L). The most frequent molecular abnormality subtypes were TEL/AML1 (*n* = 46, 18%) and hypodiploidy (*n* = 41, 16%). There were 32 (12.5%) patients who responded poorly to prednisone and 48 (18.8%) patients remained at M3 status on day 15 of induction therapy. The frequency of MRD positivity was 18.8% (*n* = 48) on day 33 and 27.7% (*n* = 71) on week 12. Thus, the standard-, intermediate-, and high-risk classifications were 25.8% (*n* = 66), 41% (*n* = 105), and 33.2% (*n* = 85), respectively.

### *GATA3* rs3824662 of pre-B-cell leukemia susceptibility

The ALL susceptibility of rs3824662 has been reported previously; however, its role in Han Chinese children remains unclear. To address this issue, we applied logistic regression analysis to investigate the association between *GATA3* rs3824662 and pre-B-cell ALL susceptibility in our study cohort. The *P*-values of the Hardy–Weinberg equilibrium in both patients and controls were >0.05, which indicated that the surveyed population had reached a genetic balance (Table 1). The A allele frequency accounted for 34.7% and 27.59% in patients with B-cell leukemia and healthy controls, respectively. The AA genotype was significantly higher in patients with pre-B-cell ALL than that in the non-ALL children (OR = 1.41, 95% CI = 1.042–1.908, *P *= 0.026; Table 1), indicating the susceptibility of *GATA3* rs3824662 in pre-B-cell leukemia. No correlation was observed between the risk allele A and other clinical features, such as sex, age, WBC count at diagnosis, and genetic abnormalities (Table 2).

**Table 1:** Comparison of *GATA3* rs3824662 between ALL patients and controls.

	RAF	Genotype count			HWP	ALL vs. non-ALL		
		CC	CA	AA		*P*	OR	95%CI
ALL cases	34.77%	109	116	31	0.98	0.026*	1.41	1.042–1.908
Controls	27.59%	89	74	11	0.39			

RAF, risk allele frequency; HWp, Hardy-Weinberg *P*-value; OR, odds ratio; CI, confidence interval.

* Significant *P*-value.

**Table 2:** The characteristics of ALL patients according to *GATA3* rs3824662.

	CC (*N* = 109)	CA (*N* = 116)	AA (*N* = 31)	*P*-value
Gender, *n* (%)				0.222
Male	68 (62.4)	66 (56.9)	14 (45.16)	
Female	41 (37.6)	50 (43.1)	17 (54.84)	
Age group, years, *n* (%)				0.925
0–10	93(85.3)	99 (85.3)	26 (83.9)	
≥10	16 (14.7)	17 (14.7)	5 (16.1)	
Initial WBC, (×10^9^/L), *n* (%)				0.77
<50	86(78.9)	93 (80.2)	23 (74.2)	
≥50	23 (21.1)	23 (19.8)	8 (25.8)	
Genetic subtypes, *n* (%)				
Hyperdiploid				0.187
Yes	17 (15.6)	22 (20.0)	2 (6.6)	
No	92 (84.4)	94 (81.0)	29 (93.4)	
TEL/AML1				0.443
Yes	23 (21.2)	17 (14.7)	6 (19.4)	
No	86 (78.9)	99 (85.3)	25 (80.6)	
E2A/PBX1				0.574
Yes	6 (5.5)	9 (7.8)	1 (3.2)	
No	103 (94.5)	107 (92.2)	30 (96.8)	
BCR/ABL				0.266
Yes	5 (4.6)	5 (4.3)	0 (0)	
No	104 (96.4)	111 (95.7)	31 (100)	
MLLr				0.446
Yes	2 (1.8)	4 (3.4)	2 (6.5)	
No	107 (98.2)	112 (96.6)	29 (93.5)	

### Effect of *GATA3* rs3824662 genotype on the treatment response

**Figure 1:**
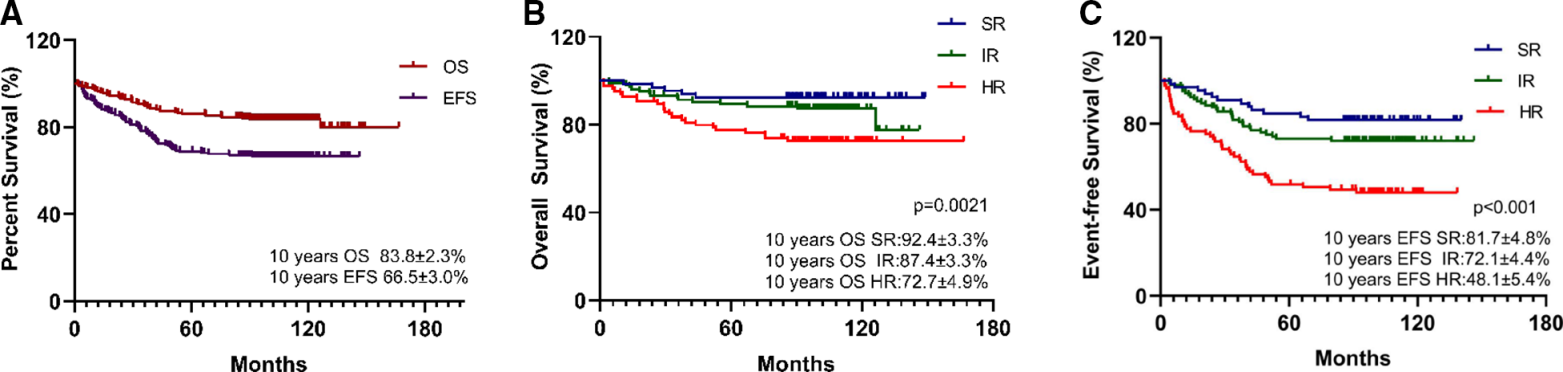
Survival analysis of pre-B-ALL treated by CCLG-2008 protocol (**A**) and the impact of risk group on long-term survival: 10-year OS (**B**), 10-year EFS (**C**), with *P*-values estimated by log-rank test (*P *= 0.0021 and *P *< 0.001), suggesting that risk group associated with poor survival.

**Figure 2:**
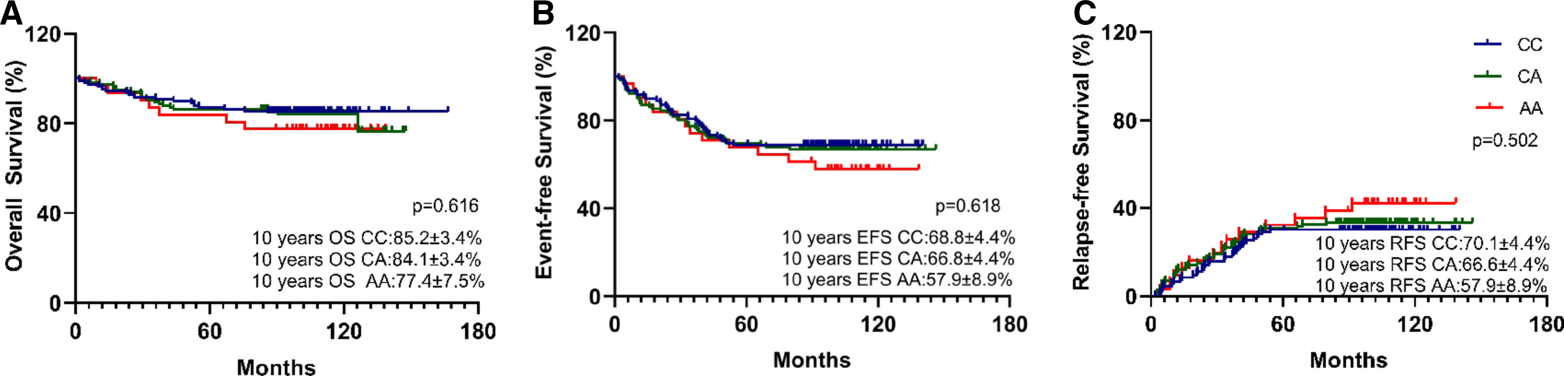
Kaplan-Meier curves for 10-year OS (**A**), 10-year EFS (**B**), and 10-year RFS (**C**) by *GATA3* SNP rs3824662 genotype (AA, AC, or CC). Differences were assessed using a log-rank test. *P*-value was 0.616, 0.618, and 0.502 respectively. The A allele has no impact on long-term survival in B-ALL.

Overall, the estimated 10-year OS, RFS, and EFS were 83.8 ± 2.3%, 66.5 ± 3.0%, and 67.4 ± 3.0%, respectively (Figure 1), and the cumulative relapse rate was 32.0% (82/256). According to risk classification, the estimated 10-year OS and EFS in SR, IR, and HR were 92.4 ± 3.3% and 87.4 ± 3.3% vs. 72.7 ± 4.9% and 81.7 ± 4.8% and 72.1 ± 4.4% vs. 48.1 ± 5.4%, respectively (Figure 1). To evaluate the influence of rs3824662 on long-term prognosis, we associated the genotype with the survival and relapse data. Patients with the AA genotype had a shorter EFS term and a higher rate of relapse than that observed in patients with other genotypes. However, no significant differences were observed between the groups. The estimated 10-year OS and EFS for the CC, CA, and AA genotypes were 85.2 ± 3.4% and 84.1 ± 3.4% vs. 77.4 ± 7.5%, and 66.8 ± 4.4% and 66.8 ± 4.4% vs. 57.9 ± 8.9%, respectively. The *P*-values were 0.616 and 0.618, respectively (Figure 2). The estimated 10-year RFS in the AA genotype was lower than that in the CC and CA genotypes (57.9 ± 8.9% vs. 66.6 ± 4.4% and 70.1 ± 4.4%, respectively, *P *= 0.370), and the distribution of relapse in the AA and CC + CA genotypes was 41.9% (13/31) and 30.6% (69/225), respectively (Table 3 and Figure 2).

**Table 3:** The effect of *GATA3* genotypes on the treatment response and outcome in pediatric ALL.

	CC (*N* = 109)	CA (*N* = 116)	AA (*N* = 31)	*P*-value
Prednisone Response, *n* (%)				0.011*
Good	97 (89.0)	105 (90.5)	22 (71.0)	
Poor	12 (11.0)	11 (9.5)	9 (29.0)	
Day 15^th^ BM, *n* (%)				0.007*
M1 + M2	89(81.7)	100 (86.2)	19 (61.3)	
M3	20 (18.4)	16 (13.8)	12 (38.7)	
Day 33^rd^ MRD, *n* (%)				0.606
<1 × 10^−3^	80(80.0)	84 (80.8)	21 (72.4)	
≥1 × 10^−3^	20 (20.0)	20 (19.2)	8 (27.6)	
Week 12^th^ MRD, *n* (%)				0.521
<1 × 10^−4^	67(69.8)	65 (68.4)	17 (58.6)	
≥1 × 10^−4^	29 (30.2)	30 (31.6)	12 (41.4)	
Risk group, *n* (%)				0.027*
Standard risk	23(21.1)	38 (32.8)	5 (16.1)	
Intermediate risk	50 (45.9)	45 (38.8)	10 (32.3)	
High risk	36 (33.0)	33 (28.5)	16 (51.6)	
Relapse, *n* (%)				0.355
Yes	31(28.4)	38 (32.8)	13 (41.9)	
No	78 (71.6)	78 (67.2)	18 (50.1)	
Relapse stage, *n* (%)				0.409
Very early stage	8(25.8)	16 (42.1)	5 (38.5)	
Early stage	9 (29.0)	8 (21.5)	1 (7.7)	
Late stage	14 (45.2)	14 (36.8)	7 (53.8)	
Survival times, SE				
10-years OS, cumulative survival	85.2 (3.4)	84.1 (3.4)	77.4 (7.5)	0.616
10-years EFS, cumulative survival	68.8 (4.4)	66.8 (4.4)	57.9 (8.9)	0.618
10-years RFS, cumulative survival	70.1 (4.4)	66.6 (4.4)	57.9 (8.9)	0.502
Adverse events, *n* (%)				
Sepsis				0.021*
Yes	48 (44.0)	52 (44.8)	22 (70.9)	
No	61 (56.0)	64 (55.2)	9 (29.1)	
Pneumonia				0.208
Yes	30 (27.5)	44 (37.9)	12 (38.7)	
No	79 (72.5)	72 (62.1)	19 (61.3)	
Myelosuppression				0.254
Yes	84 (77.1)	94 (81.0)	28 (90.3)	
No	25 (22.9)	22 (19.0)	3 (9.7)	
Liver dysfunction				0.536
Yes	19 (17.5)	25 (21.5)	8 (25.8)	
No	90 (82.5)	91 (78.5)	23 (74.2)	

BM, bone marrow; MRD, minimal residual disease detected by Flow cytometry; SE, standard error.

* Significant *P*-value.

To further investigate the effect on treatment response, we analyzed the association between rs3824662 and morphologic and MRD responses. As shown in Table 3, patients with the AA genotype had a poorer prednisone treatment response than those with the CC and CA genotypes (9/23 vs. 22/202, *P* = 0.011), as reflected by peripheral blood blast counts. On day 15 of induction therapy, the M3 status was more frequent in the AA genotype than the M1 or M2 status (12/36 vs. 19/189, *P* = 0.007; Table 3). Although a slightly high MRD level was observed in patients with the AA genotype, the difference was not statistically significant (*P *= 0.606 and *P *= 0.521, respectively; Table 3). Interestingly, the high-risk ALL patients were more frequent in the AA genotype group than that in the SR or IR patients (*P* = 0.027; Table 3). To further clarify whether the prognosis of *GATA3* SNP was affected by risk-based treatment, we analyzed the prognosis of the *GATA3* rs3824662 genotype in the same risk group. The results showed that *GATA3* rs3824662 had less influence on prognosis than the risk group did (Supplementary Figure S1).

### *GATA3* rs3824662 associated with susceptibility to sepsis

**Figure 3:**
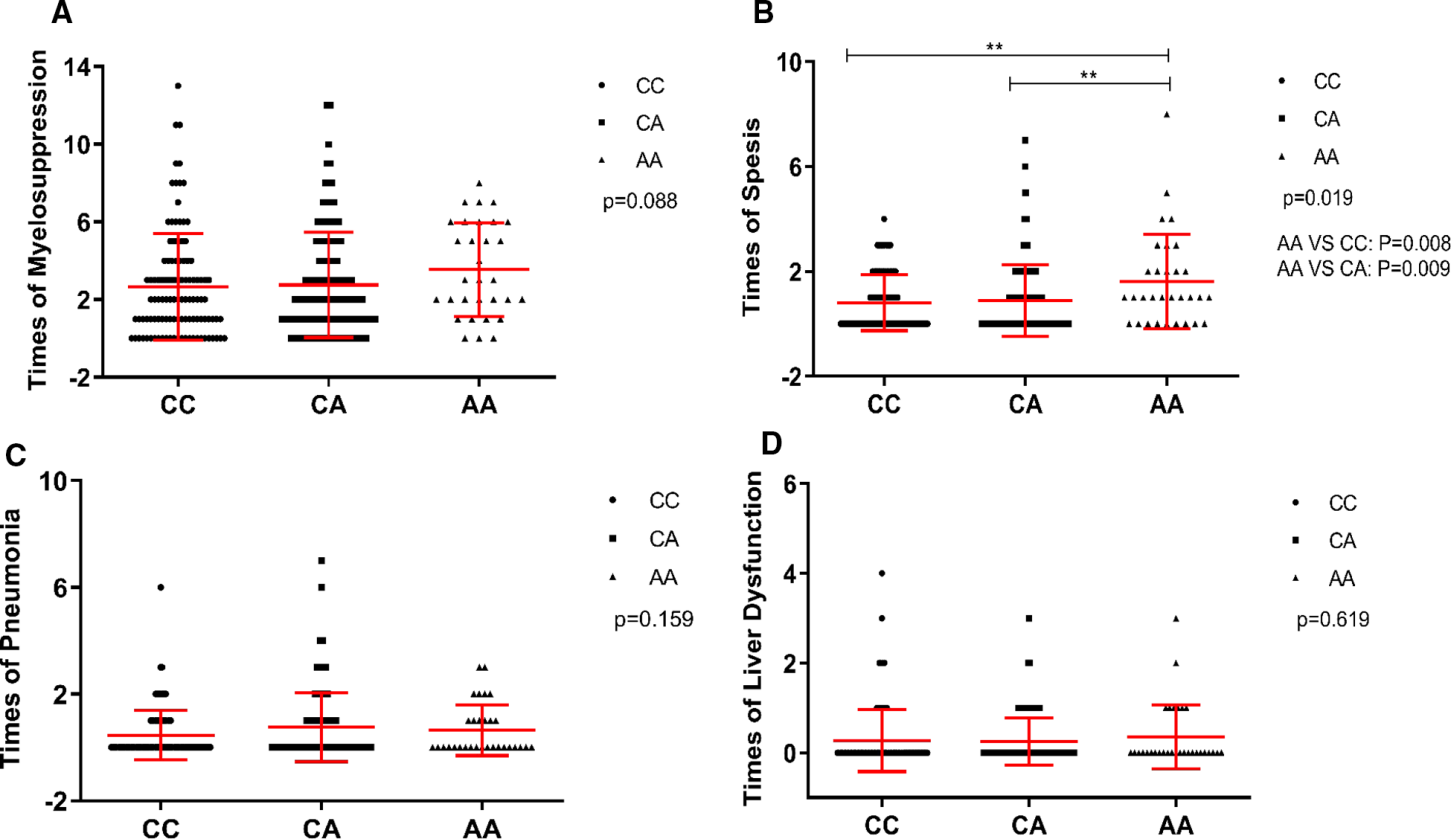
Association of times of myelosuppression (**A**), sepsis (**B**), pneumonia (**C**), and liver dysfunction (**D**) with genotype at rs3824662 in CCLG-2008 protocol, with *P*-values estimated by Kruskal-Wallis test. Indicating that the *GATA3* SNP rs3824662 genotype has sepsis susceptibility.

*GATA3* is a master transcription factor involved in immune regulation. Thus, we investigated whether rs3824662 could increase the occurrence of chemotherapy-related infections. To test this hypothesis, we performed an association analysis between the rs3824662 genotype and treatment-related adverse effects. The risk allele A was associated with susceptibility to sepsis. The AA genotype had a higher occurrence of sepsis than the other genotypes (22/31 vs. 100/125, *P* = 0.021, Table 3). In addition, some patients experienced more than one adverse event during chemotherapy. Therefore, we performed a statistical analysis of the number of adverse events. As shown in Figure 3, patients with the AA genotype had more episodes of sepsis than those with the CC or CA genotype (*P *= 0.019). However, we did not identify a significant association between the rs3824662 genotype and other side effects, such as myelosuppression, pneumonia, and liver dysfunction (Table 3 and Figure 3).

Next, we analyzed the features of sepsis among the rs3824662 genotypes. In general, based on our data, sepsis was most often caused by respiratory system infection, with rates of 85.4%, 88.5%, and 72.7% in the CC, CA, and AA genotypes, respectively. Bacteria were common pathogens in sepsis, whereas fungal infection was often accompanied by a longer course of treatment. High-risk group treatment may also have contributed to the incidence of sepsis, accounting for 43.4% of patients with sepsis. As most sepsis occurred during the period of bone marrow suppression after chemotherapy, advanced antibiotics, such as imipenem and vancomycin, were mainly used to cover most bacteria in early empirical treatment. There were no significant differences in sex, age, pathogens, length of stay, risk group, and grade of sepsis among the three groups (Table 4). Importantly, AA genotype remained statistically significantly associated with sepsis after adjusting clinical variables (i.e., age, white blood cell count, and risk group) (HR = 3.375; *P *= 0.01), as shown in Table 5.

**Table 4:** The characteristics of sepsis among *GATA3* rs3824662 genotypes in pediatric ALL.

	CC (*N* = 48)	CA (*N* = 52)	AA (*N* = 22)	*P*-value
Gender, *n* (%)				0.057
Male	30(62.5)	28 (53.8)	7 (31.8)	
Female	18 (37.5)	24 (46.2)	15 (68.2)	
Age group, years, *n* (%)				0.681
0–10	44(91.7)	45 (86.5)	19 (86.4)	
≥10	4 (8.3)	7 (13.5)	3 (13.6)	
Infection sites, *n* (%)				0.468
Respiratory system	41(85.4)	46 (88.5)	16 (72.7)	
Gastrointestinal system	2 (4.2)	3 (5.8)	4 (18.2)	
Urinary system	3 (6.2)	2 (3.8)	1 (4.5)	
Oral system	2 (4.2)	1 (1.9)	1 (4.5)	
Pathogens, *n* (%)				0.596
G− bacteria	13(27.1)	13 (25.0)	7 (31.8)	
G+ bacteria	10 (20.8)	8 (15.4)	5 (22.7)	
Fungal	2 (4.2)	3 (5.8)	3 (13.6)	
No detection	23 (47.9)	28 (53.8)	7 (31.8)	
Length of stay, days, median (range)	13 (7–25)	15 (5–33)	20 (5–45)	0.325
Grade, *n* (%)				0.102
3	44(91.6)	49 (94.2)	17 (77.2)	
4	4 (8.3)	3 (5.8)	4 (18.2)	
5	0 (0)	0 (0)	1 (4.5)	
Risk group, *n* (%)				
Standard risk	7(14.6)	14 (26.9)	2 (9.1)	0.27
Intermediate risk	18 (37.5)	20 (38.5)	8 (36.4)	
High risk	23 (47.9)	18 (34.6)	12 (54.5)	
Lymphocyte subsets at diagnosis^a^				
CD3^+^ T lym, mean (SD)	60.5 (14.0)	61.5 (8.1)	77.1 (9.8)	<0.001*
CD3^+^ CD4^+^ T, mean (SD)	29.2 (9.5)	27.5 (5.7)	40.3 (6.1)	<0.001*
CD3^+^ CD8^+^ T, mean (SD)	28.9 (9.2)	33.6 (5.6)	34.8 (6.8)	0.003*
CD4^+^/CD8^+^ ratio, median (range)	1.0(0.5–4.1)	0.7(0.3–1.8)	1.2(0.6–2.0)	0.007*
Lymphocyte subsets during sepsis^b^				
CD3^+^ T lym, mean (SD)	49.0 (14.1)	52.8 (14.2)	52.3 (14.5)	0.920
CD3^+^ CD4^+^ T, mean (SD)	23.1 (8.8)	23.0 (6.9)	22.0 (9.6)	0.698
CD3^+^ CD8^+^ T,mean (SD)	24.7 (8.9)	27.6 (9.9)	27.5 (8.6)	0.786
CD4^+^/CD8^+^ ratio, median (range)	0.9(0.3–2.0)	0.8(0.5–2.5)	0.7(0.2–2.6)	0.338

Lym, lymphocyte.

^a^ A total of 44 patients with CC genotype,46 patients with CA genotype, and 20 patients with AA genotype were included in the analysis.

^b^ A total of 20 patients with CC genotype, 18 patients with CA genotype and 12 patients with AA genotype were included in the analysis.

* Significant *P*-value.

**Table 5:** Multivariable analysis of GATA3 rs3824662 genotype for association with sepsis risk.

Patient characteristics		Hazard Ratios	95% CI	*P*-value
*GATA3* rs3824662 genotype (CC as reference)	* *			0.036*
	CA	1.175	0.676–2.044	0.567
	AA	3.375	1.333–8.548	0.010*
Age at diagnosis, years (<10 as reference)		0.373	0.171–0.812	0.013*
WBC at diagnosis, ×109/L (<50 as reference)		1.205	0.613–2.370	0.588
Risk group (SR as reference)				0.002*
	IR	1.779	0.898–3.536	0.099
	HR	3.809	1.771–8.192	0.001*

WBC, white blood cell; SR, standard risk; IR, Intermediate risk; HR, high risk.

* Significant *P*-value.

Notably, patients with AA genotype had a higher percentage of CD3^+^ T cells than patients with CC and CA genotypes, mainly represented by the higher proportion of CD4^+^ T cells (*P *< 0.001) in peripheral blood at diagnosis (Table 4 and Supplementary Figure S2). Although there was no significant difference in the percentages of lymphocyte subsets among the rs3824662 genotypes in the early stage of sepsis. Compared with CC and CA genes, patients with AA genotype showed a more significant downward trend of CD3^+^ T cells and CD4^+^ T cells from diagnosis to sepsis (Supplementary Figure S3).

## Discussion

*GATA3* rs3824662 has been described as an ALL-susceptibility locus in several pediatric pre-B-cell ALL cohorts. Recent studies have also shown its impact on diabetes and systemic lupus erythematosus because of its crucial role in immunity (14, 15). In our ALL patients, the frequency of the AA genotype was close to the frequency tested in European populations and higher than that reported in African populations (16). Consistent with previous findings, we showed that the AA genotype was associated with poor early treatment outcomes in induction therapy. In addition, for the first time, a relationship between the AA genotype and the risk of sepsis during chemotherapy was documented. However, *GATA3* rs3824662 did not show a significant difference in long-term prognosis.

Gabriele et al. identified *GATA3* rs3824662 as a novel susceptibility locus for European childhood ALL through genome-wide association studies and linked it to poor early treatment outcomes (16). Similarly, Joanna et al. verified that an intronic mutation of *GATA3* led to the risk of ALL in Polish pediatric patients, and the risk allele contributed to a high burden of MRD at the end of induction therapy (12). Consistent with the above studies, our study also revealed an increased frequency of the risk allele in pediatric pre-B-cell ALL compared to that in healthy controls (OR = 1.41, 95%, CI = 1.042–1.908, *P* = 0.026), which suggests that the A allele may be a risk factor for the development of pediatric pre-B-cell ALL in Chinese populations. The AA genotype had a poor prednisone response and a high level of bone marrow blasts on day 15 (*P* = 0.011 and 0.007, respectively), indicating its possible role in drug resistance in early chemotherapy. Therefore, *GATA3* rs3824662 may be related to leukemogenesis and drug resistance in ALL. Aberrant *GATA3* expression in different types of leukemia reveals its special role in oncogenesis. *GATA3* is conventionally used as a tumor suppressor in early T-cell precursor ALL (ETP-ALL). Inactive mutations in *GATA3,* resulting in low *GATA3* expression in ETP-ALL, are related to high DNA methylation and FLT3 mutations (17). However, a test of GATA3 mRNA expression in different types of leukemia demonstrated that compared to ETP-ALL patients, some B-ALL and non-ETP ALL patients showed extremely high GATA3 expression, suggesting that *GATA3* may also act as an oncogene (18). In NOTCH1-induced T-ALL, *GATA3* modulated the NOTCH1-enhancer by driving nucleosome eviction for the initiation and maintenance of tumors (19). However, the role of *GATA3* in B-ALL remains unclear. Qianqian et al. reported that *GATA3* may influence the development of B-ALL *via* epigenetic regulation of several pathways associated with leukemia (20). The *GATA3* rs3824662 locus was identified with significantly high expression of *GATA3* mRNA and DNase I hypersensitivity in lymphoblast cell lines (5). Moreover, PU.1 and P300 were found to bind to the rs3824662 loci in ChIp-seq data of lymphoid cells, which further confirmed the possible enhancer activity, indicating that the A allele affects local chromium access and transcription activity to improve *GATA3* expression (5). Therefore, further research is required to explore the role of *GATA3* rs3824662 in pre-B-cell ALL.

Our study demonstrated that the AA genotype of rs3824662 was associated with the occurrence of sepsis during chemotherapy. Several genetic variations in genes with immunological functions, such as *CTL4*, *IL1-B*, *IL6*, *CD40,* and *HMGB1,* have been reported to be associated with sepsis (21–25). GATA3 also plays an important role in immunology, particularly in the differentiation of Th2 cells (11). In our study, a significantly high percentage of CD3^+^ T cells, caused mainly by the high proportion of CD4^+^ T cells, was observed at diagnosis in patients with AA genotype. The transcription factor *GATA3* has emerged as a critical regulator of both innate and adaptive immunities. Several clinical studies have shown that most pediatric ALL patients have a lower proportion of CD4+ and CD8+ T cells in the peripheral blood at diagnosis than healthy children of the same age stage. After cessation of chemotherapy, defects in immunity can recover to a certain level (26–28). Although *GATA3* was originally identified as a master regulator of Th2 differentiation in CD4^+^ T cells, increasing evidence suggests that it is critical for the maintenance of mature T cells in the periphery (11). Therefore, we considered that the promotion of *GATA3* expression by the rs3824662 site could, to a certain extent, compensate for the inhibition of tumor immunity to T cells in peripheral blood, contributing to the maintenance of T cells in peripheral blood at a stable level in the early stage of leukemia. In addition, the onset and development of sepsis involve many types of immune cells and cytokines, which can change the adaptive immune response, ultimately inducing Th1 cell inhibition and Th2 cell polarization (29). Huang et al. confirmed that *GATA3* plays an important role in the early stages of sepsis. Triggered by TCR, upregulation of *GATA3* causes the Th1/Th2 ratio to skew toward Th2, which promotes immunosuppression. *GATA3* could also decrease the expression of the membrane adhesive protein annexin-A1, an anti-inflammatory protein, leading to an early imbalance of proinflammatory function (30). Although our data were limited in terms of Th cell subsets, risk genotype AA showed a more significant downward trend in the proportion of CD3^+^ T cells and CD4^+^ T cells from diagnosis to sepsis. Therefore, we hypothesized that the risk allele may accelerate the differentiation of Th2 cells and inhibit Th1 cells by increasing the expression of *GATA3*, ultimately leading to the balance disorder of Th1/Th2, which is more likely to cause sepsis. The *GATA3* SNP rs3824662 may have also contributed to the apoptosis of CD4^+^ T cells in the onset of sepsis, accelerating the progress of sepsis. However, relevant mechanisms still need further research.

In summary, we identified risk loci at rs3824662 for pre-B-cell ALL in Han Chinese individuals, which could be a possible risk factor for poor early treatment response and sepsis. Further studies are needed to elucidate its role in the pathogenesis of pre-B-cell ALL and in the high incidence of sepsis. These analyses will help to provide a theoretical basis for evaluating clinical prognosis and risk stratification, which will also provide molecular targets for the prevention and treatment of chemotherapy-related complications.

## Data Availability

The original contributions presented in the study are included in the article/**Supplementary Material**, further inquiries can be directed to the corresponding author/s.
