# Supplementary material for: Effect of GATA3 rs3824662 gene polymorphism in Han Chinese children with pre-B-cell acute lymphoblastic leukemia with 10 years follow-up
Source: Front Pediatr. 2023 Jan 11;10:1044866. doi: 10.3389/fped.2022.1044866 (PMC9875006; doi:10.3389/fped.2022.1044866)
Supplement: Supplementary file 1 [file Datasheet1.pdf]

Supplementary Table 1 Baseline characteristics of patients

	Pediatric ALL (N=256)
Gender, n (%)	
Male	148(57.8)
Female	108(42.2)
Age at diagnosis, years; n (%)	
0-10	218(85.2)
≥ 10	38(14.8)
Initial WBC, ($x10^9/L$), n (%)	
< 50	202(78.9)
≥ 50	54(21.1)
Genetic subtypes, n (%)	
Hyperdiploid	41(16.0)
Hypodiploid	1(3.9)
TEL/AML1	46(18.0)
E2A/PBX1	16(6.3)
BCR/ABL	10(3.9)
MLLr	8(3.1)
B-other	130(50.8)
NA	4(15.6)
Prednisone Response, n (%)	
Poor	32(12.5)
Good	224(87.5)
$D15^{th}$ BM blast, n (%)	
M1+M2	208(81.2)
M3	48(18.8)
Day 33rd MRD, n (%)	
$<1X10^{-3}$	184(71.9)
$\geq 1X10^{-3}$	48(18.8)
Week 12th MRD, n (%)	
$<1X10^{-4}$	148(57.8)
$\geq 1X10^{-4}$	71(27.7)
Risk group, n (%)	
Standard risk	66(25.8)
Intermediate risk	105(41.0)
High risk	85(33.2)
Relapse stage, n (%)	
Very early stage	29(11.3)
Early stage	18(7.0)
Late stage	35(13.7)
Survival times, SE	
10-years OS, cumulative survival	83.8±2.3

10-years EFS, cumulative survival 66.5±3.0

10-years RFS, cumulative survival 67.4±3.0

WBC:white blood cell;BM: bone marrow;MRD: minimal residual disease detected by Flow cytometry; SE: standard error.

Supplementary Figure 1. Kaplan-Meier curves for 10-year OS (A-C) and 10-year EFS (D-F) by *GATA3* SNP rs3824662 genotype (AA, AC, or CC) in the same risk group. Differences are assessed using a log-rank test. Suggesting not rs3824662 genotype but risk group has poor impact on long-term prognosis in pre-B cell ALL in CCLG-2008 protocol.

Supplementary Figure 2. Association of the *GATA3* SNP rs3824662 genotype with lymphocyte subsets at diagnosis. The percent of $CD3^+$ T cell, $CD4^+$ T cell and $CD8^+$ T cell in peripheral blood is shown in A-C. Data shown are mean±SD. Multiple comparisons were evaluated by 1-way ANOVA with Turkey's multiple-comparison test. *P < 0.05; ***P < 0.001.

Supplementary Figure 3. The trend of lymphocyte subsets from diagnosis to the onset of sepsis by *GATA3* SNP rs3824662. The percent of $CD3^+$ T cell, $CD4^+$ T cell and $CD8^+$ T cell in peripheral blood is shown in A-C. Comparison of paired data were evaluated by paired T-test. *P < 0.05; **P < 0.01;*P < 0.001.**
