# Supplementary figures and images for: Effect of GATA3 rs3824662 gene polymorphism in Han Chinese children with pre-B-cell acute lymphoblastic leukemia with 10 years follow-up
Source: Front Pediatr. 2023 Jan 11;10:1044866. doi: 10.3389/fped.2022.1044866 (PMC9875006; doi:10.3389/fped.2022.1044866)

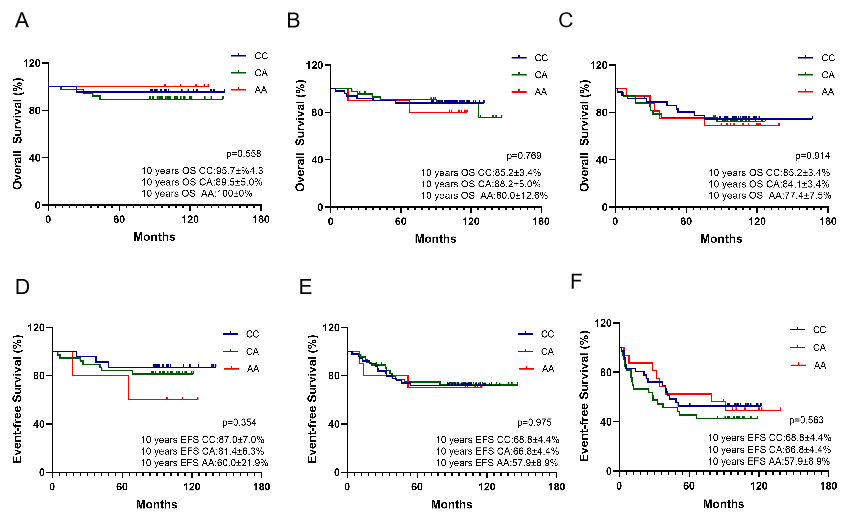

Supplement: Supplementary file 2 [file Image1.tif]

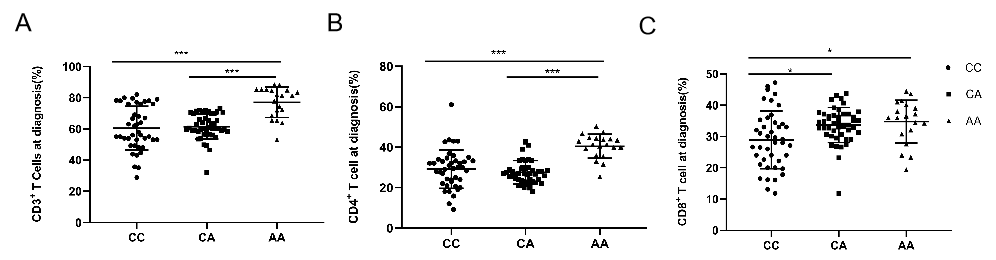

Supplement: Supplementary file 3 [file Image2.tif]

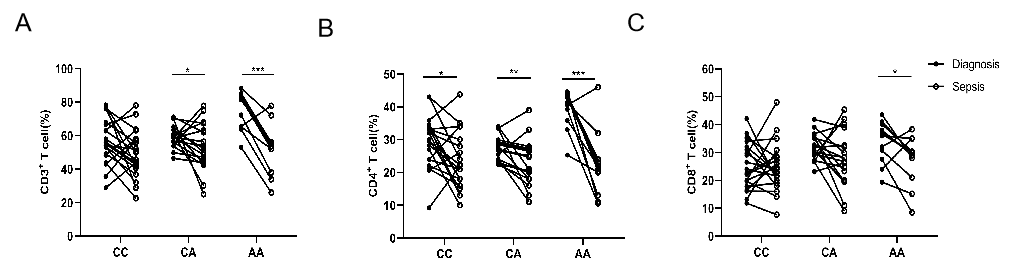

Supplement: Supplementary file 4 [file Image3.tif]
